# Tribological Aspects Concerning the Study of Overhead Crane Brakes

**DOI:** 10.3390/ma15196549

**Published:** 2022-09-21

**Authors:** Miorita Ungureanu, Nicolae Medan, Nicolae Stelian Ungureanu, Nicolae Pop, Krzysztof Nadolny

**Affiliations:** 1 North University Centre at Baia Mare, Faculty of Engineering, Technical University of Cluj-Napoca, V. Babes Str. 62A, 430083 Baia Mare, Romania; 2 Institute of Solid Mechanics of Romanian Academy, Constantin Mille Str. 15, 010141 Bucharest, Romania; 3 Department of Production Engineering, Faculty of Mechanical Engineering, Koszalin University of Technology, Racławicka Str. 15–17, 75-620 Koszalin, Poland

**Keywords:** brake lining, coefficient of friction, design of experiment, frictional contact, multilevel factorial plan, tribology, wear

## Abstract

The aim of the study is the tribological analysis of the crane drum brakes. A theoretical analysis of the wear processes for brake lining was performed and the coefficient of friction under tribological conditions was determined experimentally simulating the operating conditions for three types of brakes. The theoretical study of the wear was oriented towards of determining the lifetime of the brake lining. In the experimental determination of the coefficient of friction, the following parameters were taken into account: the contact pressure between the shoe and the drum; the initial speed of the brake drum; the humidity of the working environment; and the temperature of the drum-brake lining friction surfaces. After performing the experiments, a statistical analysis was conducted, that shows the amount the coefficient of friction is influenced by the previously mentioned parameters: the highest weight was humidity with a value of 35.58%, followed by temperature with a percentage of 23.95%, velocity with 4.54%, and lastly pressure with 4.19%. Furthermore, the equation that expresses the dependence between the coefficient of friction and the parameters is determined. We consider that the results obtained are important for brake manufacturers in order to improve braking efficiency and the safety of overhead cranes.

## 1. Introduction

Overhead cranes are equipped with various types of brakes, which operate in different conditions. For this reason, a detailed analysis is needed regarding the kinematics of motion, the evolution of motion over time, the characteristics of the tribological stresses and the environmental conditions (humidity, temperature) for different cases.

The drum of the brakes of overhead cranes is made from steel or cast iron and the brake lining of the shoe is made from composite materials [1]. The material, from which the brake drum is made, must have the following characteristics from a tribological point of view: high coefficient of friction; stable coefficient of friction between itself and the brake lining; wear resistance; good thermal conductivity [2].

The main features required of the brake lining are: high coefficient of friction between itself and the brake drum, wear resistance, tensile strength, good characteristics on cooling, stable friction levels [3,4,5]. Mechanical and thermal stresses, as well as some physico-chemical effects, can destroy or restore the micro-geometry of surface layers [6]. At the level of the composite material, the friction force has two components, namely: adhesion and deformation. Adhesion is determined by van der Waals forces, bipolar interactions, hydrogen bonds, and electric charge. Deformations are due to: deformation of the roughness of the materials, loss of energy due to hysteresis and grooving [7]. The hysteresis depends on contact pressure, strain rate and temperature and the viscoelastic effects determine the dependence between the coefficient of friction and the temperature [8,9].

The set of conditions straining the pair of drum-brake lining, viewed from a tribological point of view, leads to wear. Wear in this context is the result of the material interactions of the surfaces in contact [10].

Maintaining a high and constant coefficient of friction for different operating conditions requires knowledge of the practical aspects of the tribological behavior of the friction pair.

The durability of the brake lining and the brake drum depends on their wear resistance [5,6,7]. Wear of the brake lining-drum pair affects the quality of braking and leads to failure.

Numerous studies in the field have shown the dependence of the coefficient of friction, not only on the two materials in contact, but also on several tribological aspects [6,11,12]. Thus, the operating conditions and the operational parameters that influence the coefficient of friction are surface temperature, the humidity of the friction surfaces, the relative velocity of the friction pair, and the contact pressure of the friction pair [6,13,14].

The temperature of the surfaces of the friction pair influences the coefficient of friction [15]. Brake heating has a negative influence for braking quality [16]. Changes in the coefficient of friction simultaneous to temperature changes are owed to certain aspects regarding the changes which happen on a structural level within the friction pair [15].

## 2. Theoretical Considerations Regarding the Wear of the Brake Lining

Most of the studies related to these tribological aspects of shoe brakes are with reference to automobiles, trains, and lifting machines [2,3,17,18,19,20]. Regarding the shoe brakes of overhead cranes, the temperature of the friction pair, the braking dynamics, and the control of the movement in the braking process have been studied by several authors [21,22,23,24]. To determine the tribological behavior of the materials that make up the pair of friction, it is necessary to study each particular case separately, thus we carried out, for the first time, this study regarding the overhead cranes manufactured in Romania by U.M.T. Timisoara.

The tribological phenomena in the case of the brake friction pair are numerous, but we would like to specify that the present study did not address all these aspects, and instead, it was oriented in two directions, which are: the theoretical study of the wear of the brake linings; and the analysis of the variation in the coefficient of friction according to tribological factors.

The theoretical study of the wear of brake linings has as its objective the evaluation of the life of the brake lining according to the intensity of wear.

The analysis of the variation in the friction coefficient for the friction pair of cranes in relation to temperature, roughness, and contact pressure was studied by a number of authors [1,5,9,14,20]. However, they did not include overhead cranes, or more specifically, a study aimed at the simultaneous analysis of the parameters: relative speed; contact pressure and temperature on the coefficient of friction; and the weight of influence of these factors on the coefficient of friction were never looked into in these studies. This is why our research has been focused in this direction.

During the braking process, the two elements of the friction pair (brake lining–drum) wear out after several braking cycles. Assuming that the braking pair and the specific wear are constant for the given operating conditions (roughness, relative velocity of the two surfaces, etc.) the wear distribution of the shoe friction lining on the shoe-drum contact spring will be studied. The wear intensity of the friction pair has high values during the break-in period compared to the normal operation period [16,25]. As demonstrated by research in the field of brakes, where the force developed by the actuation mechanism is a basic parameter, after break-in the intensity of brake lining wear becomes uniform [16,19].

The volume of material removed for a single passage on the surface of the brake lining in the form of wear particles (∆ν) is proportional to the contact area and is calculated according to the Equation (1) [16]:

(1) $$∆\nu =∆h\cdot l_{f}\cdot b$$

where:

∆ν—volume of material worn for a single pass (for a single rotation of the drum);

∆h—average thickness removed by wear, for a single pass (for a complete rotation of the drum);

$l_{f}$—friction length for a complete rotation of the brake drum;

b—the width of the shoe.

Under these conditions, for braking once the volume of worn material is calculated according to the Equation (2):

(2) $$∆V_{o}=n\cdot ∆h\cdot l_{f}\cdot b$$

where n is the number of rotations of the drum for braking once.

In another aspect, due to the total friction length $L_{f}$ while the brake lining is operating, the thickness ∆H and volume ∆V were removed in the form of wear.

The process of wearing the brake lining can also be characterized by specific wear, as follows the Equation (3) [16]:

(3) $$U_{s}=\frac{∆V}{F_{n}\cdot L_{f}}=\frac{S\cdot ∆H}{F_{n}\cdot L_{f}}$$

where $F_{n}$—is the normal pressing force of the shoe on the drum and *S* is the surface of the shoe.

The thickness of the layer of worn material depending on the specific wear and depending on the contact pressure between the shoe and the drum (*p*) can be written as follows:

(4) $$∆H=p\cdot U_{s}\cdot L_{f}$$

and:

(5) $$L_{f}=N_{f}\cdot n\cdot l_{f}$$

where $N_{f}$ is the number of brakes for the lifespan of the brake lining.

If the maximum thickness of the worn material layer is known, the following calculation formula shall be obtained for Equations (3) and (4) for the total number of brakes ($N_{f}$) during the lifespan of the brake lining:

(6) $$N_{f}=\frac{∆H}{p\cdot U_{s}\cdot n\cdot l_{f}}$$

**Figure 1:**
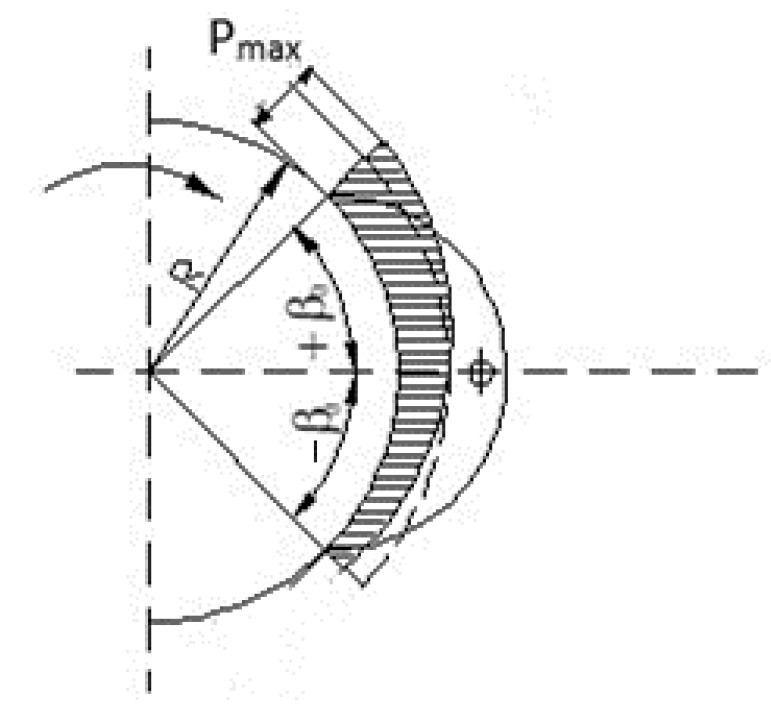
Shoe-drum contact pressure distribution for an articulated fixed shoe from lifting installations [16].

Regarding the contact pressure between the shoe and the drum, research in the field has shown that, for shoe brakes, the contact pressure is maximum at the end of the shoe coming into contact with the drum, and the minimum is recorded at the end of the shoe coming out of contact with the drum [16] (Figure 1).

The contact pressure between the shoe and the drum in the case of an articulated fixed shoe has a distribution shown in Figure 1 [16] and is calculated as follows:

(7) $$p=\frac{M_{f}}{2\cdot \mu \cdot R^{2}\cdot b\cdot sin\beta_{0}}\left(cos\beta +\frac{sin\beta }{k}\right)$$

where:

$M_{f}$—is the moment of braking;

μ—is the coefficient of friction between the shoe brake lining and the drum;

k—is a constant that depends on the type of brake;

R—is the radius of the brake drum.

$-\beta_{0}$—is the contact angle on the middle of the shoe when it comes out of contact with the drum and

$+\beta_{0}$—is the contact angle on the middle of the shoe when it comes into contact with the drum.

Replacing the expression of the contact pressure in Equation (4) results in the equation of the thickness of the worn layer on the arc between the shoe and the drum, for the articulated fixed shoe of the shoe brake:

(8) $$∆H=\frac{U_{s}\cdot L_{f}\cdot M_{f}}{2\cdot \mu \cdot R^{2}\cdot b\cdot sin\beta_{0}}\left(cos\beta +\frac{sin\beta }{k}\right)$$

Note that the thickness of the worn brake lining material depends on: the specific wear of the brake lining; friction length; braking moment; the position (angle β) of the point considered on the shoe-drum contact arc; k which is a constant that depends on the construction of the brake.

Therefore, for β=0 result:

(9) $$∆H=∆H_{0}=\frac{U_{s}\cdot L_{f}\cdot M_{f}}{2\cdot \mu \cdot R^{2}\cdot b\cdot sin\beta_{0}}$$

Under these conditions, the thickness of the layer of worn material at a certain point located on the contact arc, characterized by the angle between the direction of the contact pressure and the horizontal axis, can be written as follows:

(10) $$∆H=∆H_{0}\left(cos\beta +\frac{sin\beta }{k}\right)$$

**Figure 2:**
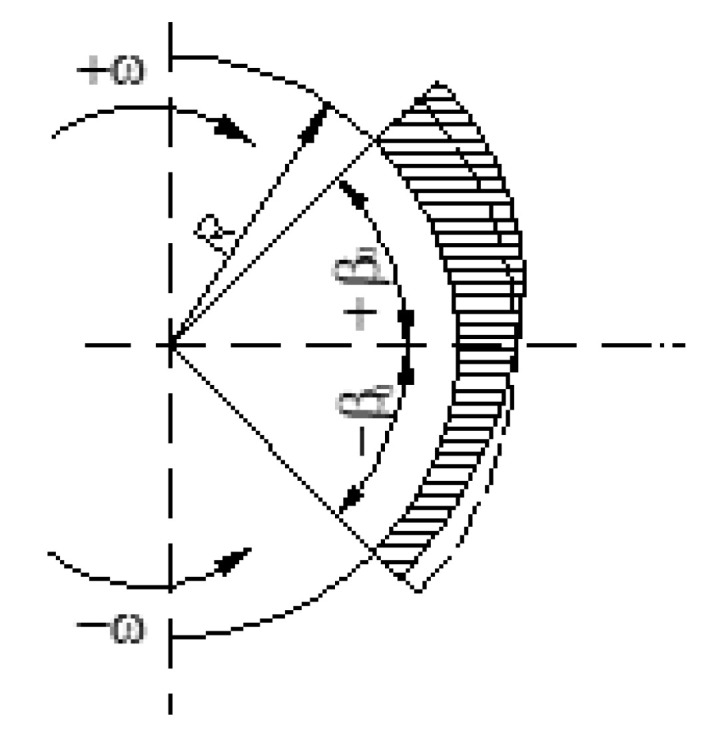
Distribution of wear on the surface of the shoe [16].

The wear distribution of the brake lining on the shoe-drum contact arc is identical to the distribution of the contact pressure, assuming that the roughness of the brake drum is constant over the entire surface. This distribution changes depending on the direction of rotation of the drum (Figure 2).

Due to the fact that while operating the brakes of lifting systems the number of brakes in one direction of rotation of the drum is approximately equal to the number of brakes in the opposite direction, the wear distribution of the brake lining is uniform to some extent, but it has two areas of maximum at the ends of the shoes, and the minimum area is in the center of the shoe.

As a conclusion of this part of the study, we would like to highlight as a novelty the evaluation method of the total number of brakes ($N_{f}$) during the lifespan of the brake lining with Equation (6). The users of the overhead cranes during use have the possibility to measure the thickness of the brake lining at the end of the shoe, to replace the value obtained in Equation (6) and to determine the number of brakes performed up to the respective date. Considering that the number of brakes during the entire lifetime of the brake lining is known they can estimate how long the brake lining can be used.

## 3. Experimental Determination of the Coefficient of Friction under Tribological Conditions for Brake Shoes within the Overhead Crane

The aim of this experiment is to determine the coefficient of friction between the brake lining and the drum when operating conditions are simulated.

Maintaining a high and constant coefficient of friction for different operating conditions requires knowledge of the practical aspects of the tribological behavior of the friction pair.

**Figure 3:**
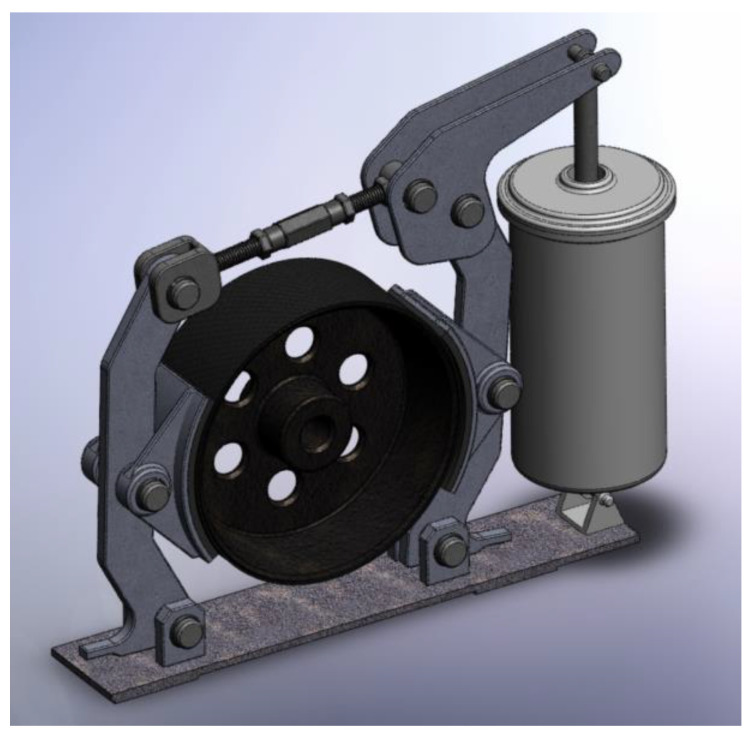
Brake shoes type FC 400 [27].

In this study, the brake shoes of the following type-dimensions were tribologically analyzed: brake type FC 200, brake type FC 315, and brake type FC 400 [26], manufactured at U.M.T. Timisoara Romania. Figure 3 shows the model of a brake type FC 400. The drum of these brakes is made of steel, and the brake shoes are covered with brake linings of composite materials type FF-30. FF-30 is environmentally friendly, with good wear resistance, a good friction characteristic and a good mechanical strength [26]. Under these conditions, the operational parameters specific to these types of brakes that form a part of overhead cranes were taken into account for the experiments.

The initial braking velocity has an influence on the heat resulting from friction and on the coefficient of friction [28]. Contact pressure also influences the coefficient of friction, and so do temperature and humidity [29].

Thus, the two components of the friction pair were subjected to compressive stress, velocity, working temperature and ambient humidity. Experiments were performed to determine the coefficient of friction in case of dry friction in relation to sliding velocity, contact pressure, temperature and humidity conditions.

**Figure 4:**
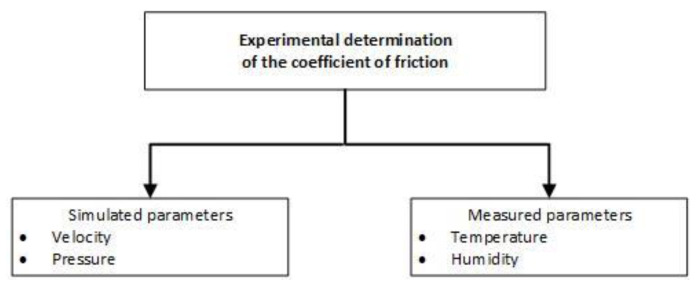
Scheme of the parameters that influence the friction coefficient.

In Figure 4, the synthesized scheme of the parameters that influence the friction coefficient considered in the experiment is shown.

**Figure 5:**
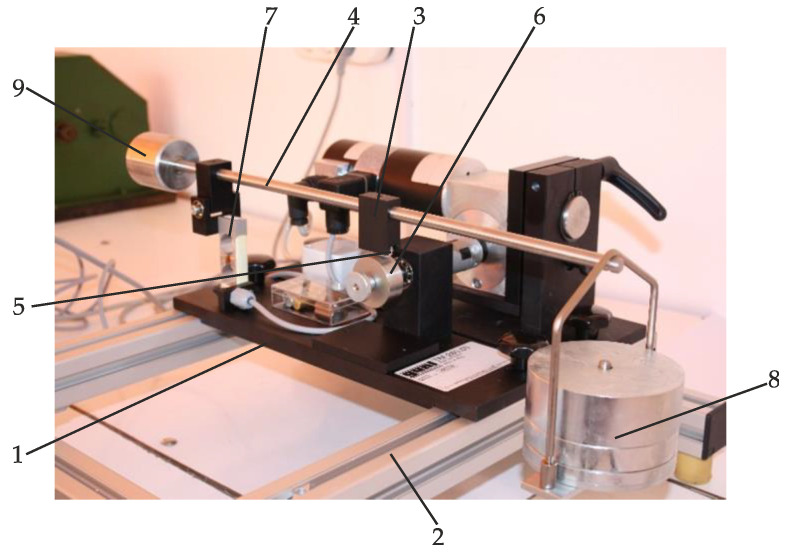
Experimental laboratory stand.

In order to determine the coefficient of friction, the laboratory stand TM260.05 (Figure 5) from the Faculty of Engineering was used. The stand was manufactured by G.U.N.T. Gerätebau GmbH Germany. The stand error is 2%.

The principle of operation and the component parts of the stand are presented in Figure 5. The module board (1), of the basic unit (2), the assay sample holder (3) adjusted and fastened on the loading arm (4). The loading arm (4) makes it possible to obtain a transmission ratio of 2:1. The scope of supply includes assay sample (5) made of three different materials. The friction wheel (6) is made of hardened stainless steel. Its surface is polished. The force transducer (7) is used to measure the friction. The set of weights (8) is used to adjust the load gradually. The counterweight (9) compensates for the unloaded weight of the loading arm (4).

The assay sample was made from FF30 brake lining and the drum from steel.

The loads applied to the assay sample were 5 N, 10 N, and 20 N, respectively, and the values of contact pressure between the drum and the assay sample were: 0.25 MPa; 0.5 MPa; 1 MPa. The tests were performed for four peripheral drum velocities: 12 m/min; 15 m/min; 18 m/min; 24 m/min.

Firstly, an atmosphere with a humidity of 60% was created within the laboratory, followed by an atmosphere with a humidity of 90% in the next stage.

The resulting values for the coefficient of friction and the temperature of the drum are presented in the Table 1, Table 2 and Table 3. The temperature of the friction surfaces was measured with a TMTL 260 thermometer, and the humidity of the environment was measured with a hygrometer TESTO 615.

**Figure 6:**
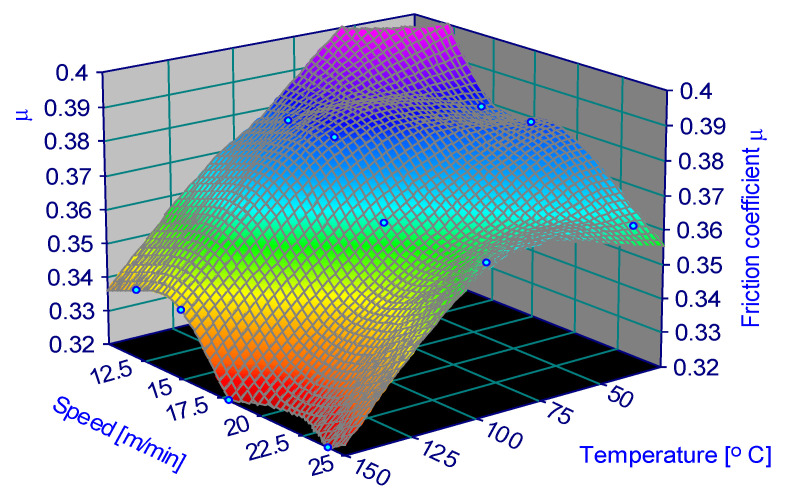
Coefficient of friction for 0.25 MPa and 60% humidity.

**Figure 7:**
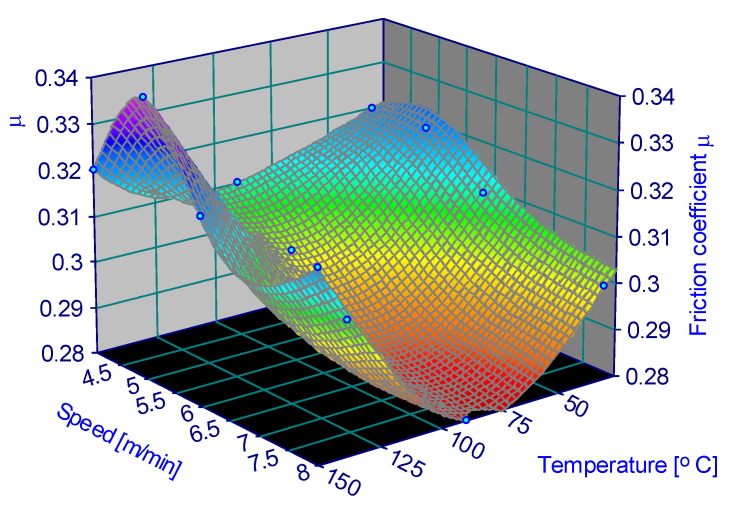
Coefficient of friction for 0.25 MPa and 90% humidity.

**Figure 8:**
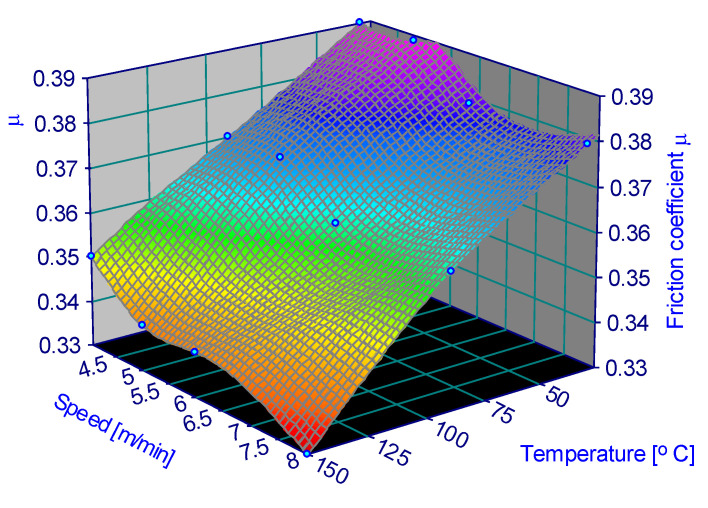
Coefficient of friction for 0.5 MPa and 60% humidity.

**Figure 9:**
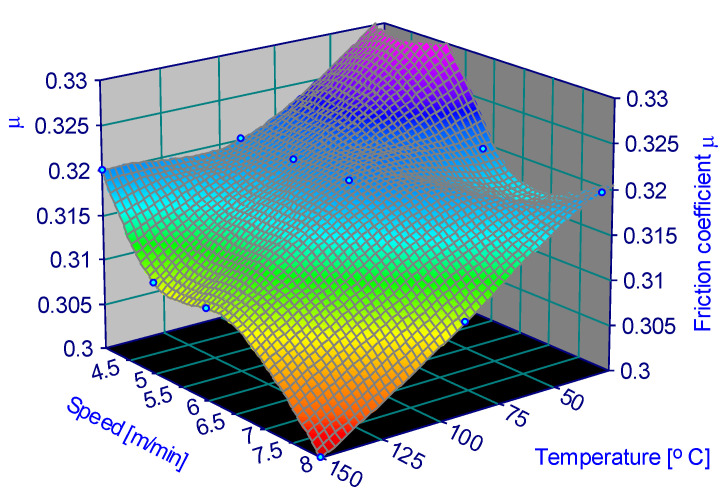
Coefficient of friction for 0.5 MPa and 90% humidity.

**Figure 10:**
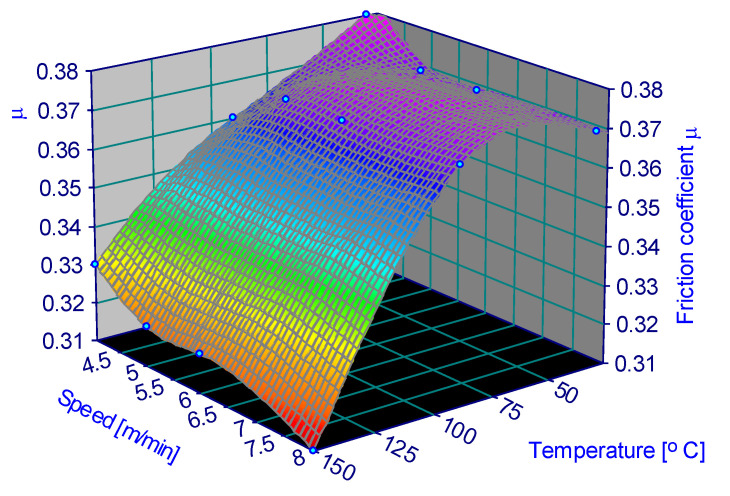
Coefficient of friction for 1 MPa and 60% humidity.

**Figure 11:**
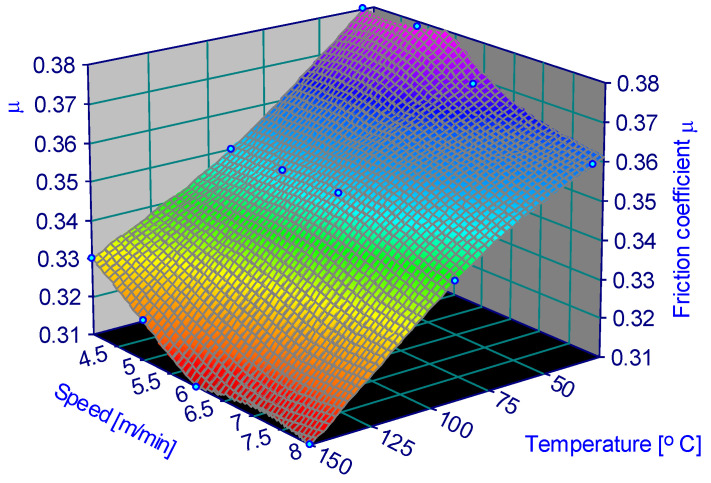
Coefficient of friction for 1 MPa and 90% humidity.

The variation in the friction coefficient depending on the parameters is represented in Figure 6, Figure 7, Figure 8, Figure 9, Figure 10 and Figure 11.

## 4. Analysis of the Experimental Data

To be able to perform statistical analysis of experimental data, it is necessary to use an experimental plan [30,31].

Given that four parameters are used, each with a different number of levels, the chosen experimental plan is the multilevel factorial plan.

In fact, Table 1, Table 2 and Table 3 present the parameters, the levels of each, and the coefficient of friction values obtained following the experiments according to a multilevel factorial plan. To summarize, for an easier view, the data are centralized in Table 4.

### 4.1. The Main Effect of Parameters

Based on the established factorial plan and using the Minitab software, the influence of the parameters on the coefficient of friction can be determined.

**Figure 12:**
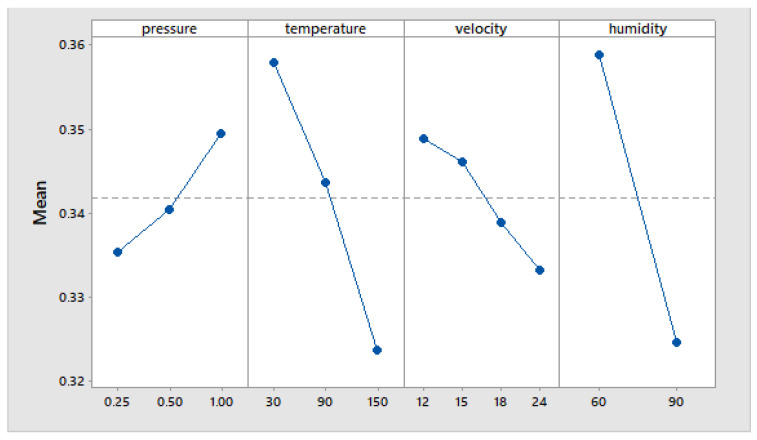
Main effect plot of the parameters on the coefficient of friction.

Figure 12 shows the main effects plot for means of the parameters on the coefficient of friction.

In Figure 12 it can be observed that for a single parameter—the pressure—an increase determines the increase in the friction coefficient.

For the other three parameters—temperature, velocity, humidity—an increase in their values determines the decrease in the coefficient of friction.

If these results are compared with other studies carried out in the field, it is found that the variation in the coefficient of friction with temperature, speed, and pressure, for the studied interval, is similar [1,28].

For a more detailed characterization of the influence of the parameters on the coefficient of friction, we suggest as the next step the determination of the weight of influence of the parameters and second-degree interactions of them.

### 4.2. The Contribution of Parameters and Their Interactions

To determine the value of the parameters’ influence and that of the second-degree interactions between them on the coefficient of friction, an ANOVA was performed. The results obtained are presented in Table 5.

**Figure 13:**
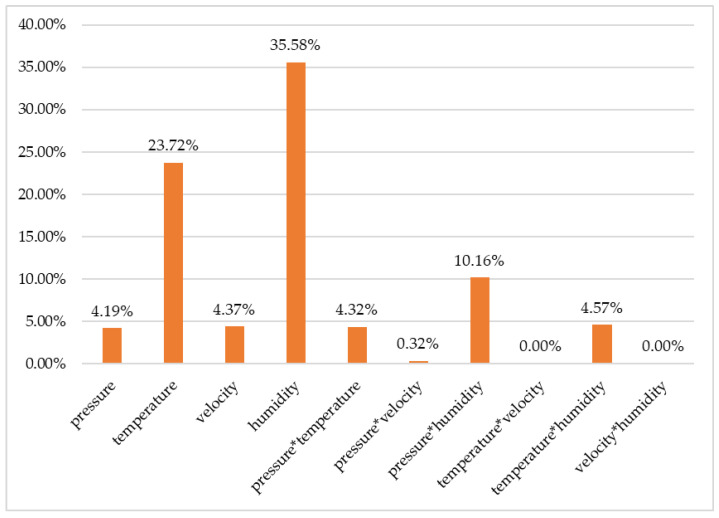
The percentage of influence of the parameters and their interactions on the coefficient of friction.

Based on Table 5, Figure 13 shows the percentage of influence, which the parameters and their second-degree interactions had on the coefficient of friction.

It can be observed that the parameter with the greatest influence on the coefficient of friction is humidity, with a value of 35.58%, followed by temperature, with a percentage of 23.95%, velocity with 4.54%, and lastly pressure with 4.19%.

Regarding the interactions between factors, the highest value has a pressure–humidity interaction with a value of 10.89%, followed by temperature–humidity with 7.28% and pressure–temperature with a value of 5.55%. Temperature–velocity and velocity–humidity interactions have an insignificant influence.

The knowledge of these variations in the coefficient of friction, depending on the parameters, is important for the brake manufacturer, giving them the possibility of making composite materials for the brake lining with properties appropriate to the operating conditions.

### 4.3. Regression Equation for the Coefficient of Friction

Based on the experimental data obtained, an equation is determined, which describes the coefficient of friction’s value depending on the parameters and the interactions between them (previously established).

In the first stage, all the parameters and all the second-degree interactions between them are considered. Table 6 shows the results obtained from ANOVA for regression analysis.

From Table 6, it can be seen that the second-degree interaction between pressure–velocity has a value of 0.32% and the second-degree interactions of temperature–velocity and temperature–humidity have a value of zero.

Given this fact, when determining the regression equation, the three interactions are excluded.

Based on the results obtained in Table 6, Equation (11) of coefficient of friction was determined.

(11) $$\begin{array}{l} Friction\;coeff=0.5863-0.0925\times p-0.000862\times t-0.001349\times v-\\ -0.003028\times h-0.00039\cdot p\times t+0.001952\times p\times h+0.000008\times t\times h \end{array}Friction\;coeff=0.5863-0.0925\times p-0.000862\times t-0.001349\times v-0.003028\times h-0.00039\cdot p\times t+0.001952\times p\times h+0.000008\times t\times h$$

The great advantage of such an equation, obtained from an ANOVA analysis resulting from a previously established experimental plan, is that the value of the coefficient of friction can be determined for any values of the parameters (even values that were not taken into account during the experiments), provided that those values of the parameters are within the established experimental domain.

An important observation is that the equation is valid only for parameter values that are contained within the established experimental domain (Table 4).

## 5. Conclusions

For the theoretical analysis of the wear of brake linings for functioning the brake shoe, we have determined an evaluation method of the total number of brakes ($N_{f}$) during the lifespan of the brake lining. This method is important for users of overhead cranes.

Another important contribution of this study is the experimental determination of the coefficient of friction in tribological conditions for the brake shoes of overhead cranes. For this, the experiments were carried out depending on the influence of four parameters: the contact pressure between the shoe and the drum; the initial velocity of the drum; the temperature of the friction surfaces; and ambient humidity. Analyzing the results obtained from the experiments and from processing the experimental data it can be observed that the coefficient of friction decreases with increasing temperature, humidity, and the sliding velocity between the two elements of the friction pair. It can also be noted that the coefficient of friction increases with increasing contact pressure.

Starting from the established experimental range and using statistical analysis, a regression equation was obtained, that would allow the determination of the value of the coefficient of friction for any value of the four established parameters, but with the condition that the values are included in the established experimental range. It was learned that the weight of the influence of the parameters on the coefficient of friction was as follows: humidity with a value of 35.58%; followed by temperature with a percentage of 23.95%; velocity with 4.54%; and contact pressure with 4.19%.

The presented methods allow for a better evaluation of the coefficient of friction in the tribological operating conditions and give the possibility to estimate the service life for the brake linings of the brake shoes from overhead cranes.

The present paper does not and cannot pretend to cover the entire subject matter, with the field being particularly complex. The theoretical and experimental research conducted had the goal of finding solutions to at least some of the problems that appear in this case.

In the future we aim to continue this study with the experimental determination of the wear intensity in tribological conditions for the friction pair and the study of the deformations of the friction pair elements.

## Figures and Tables

**Table 1:** Coefficient of friction and temperature for 0.25 MPa.

Pressure [MPa]	Temperature [°C]	Velocity [m/min]	Humidity [%]	Coefficient of Friction
0.25	30	12.00	60	0.40
			90	0.32
		15.00	60	0.38
			90	0.32
		18.00	60	0.38
			90	0.31
		24.00	60	0.36
			90	0.30
	90	12.00	60	0.38
			90	0.31
		15.00	60	0.38
			90	0.30
		18.00	60	0.36
			90	0.29
		24.00	60	0.36
			90	0.28
	150	12.00	60	0.34
			90	0.32
		15.00	60	0.34
			90	0.34
		18.00	60	0.32
			90	0.32
		24.00	60	0.32
			90	0.32

**Table 2:** Coefficient of friction and temperature for 0.5 MPa.

Pressure [MPa]	Temperature [°C]	Velocity [m/min]	Humidity [%]	Coefficient of Friction
0.5	30	12.00	60	0.39
			90	0.33
		15.00	60	0.39
			90	0.33
		18.00	60	0.38
			90	0.32
		24.00	60	0.38
			90	0.32
	90	12.00	60	0.37
			90	0.32
		15.00	60	0.37
			90	0.32
		18.00	60	0.36
			90	0.32
		24.00	60	0.36
			90	0.31
	150	12.00	60	0.35
			90	0.32
		15.00	60	0.34
			90	0.31
		18.00	60	0.34
			90	0.31
		24.00	60	0.33
			90	0.30

**Table 3:** Coefficient of friction and temperature for 1 MPa.

Pressure [MPa]	Temperature [°C]	Velocity [m/min]	Humidity [%]	Coefficient of Friction
1	30	12.00	60	0.38
			90	0.38
		15.00	60	0.37
			90	0.38
		18.00	60	0.37
			90	0.37
		24.00	60	0.37
			90	0.36
	90	12.00	60	0.36
			90	0.35
		15.00	60	0.37
			90	0.35
		18.00	60	0.37
			90	0.35
		24.00	60	0.37
			90	0.34
	150	12.00	60	0.33
			90	0.33
		15.00	60	0.32
			90	0.32
		18.00	60	0.32
			90	0.31
		24.00	60	0.31
			90	0.31

**Table 4:** The values of the parameters for Full factorial method.

Parameter	Values
Pressure [MPa]	0.25; 0.5; 1
Temperature [°C]	30; 90; 150
Velocity [m/min]	12; 15; 18; 24
Humidity [%]	60; 90

**Table 5:** ANOVA to determine the contribution of parameters and their interactions.

Source	DF (Degree of Freedom)	Seq SS	Contribution [%]
pressure	2	0.002478	4.19
temperature	2	0.014144	23.95
velocity	3	0.002682	4.54
humidity	1	0.021012	35.58
pressure × temperature	4	0.003281	5.55
pressure × velocity	6	0.000356	0.60
pressure × humidity	2	0.006433	10.89
temperature × velocity	6	0.000089	0.15
temperature × humidity	2	0.0043	7.28
velocity × humidity	3	0.000037	0.06
Error	61	0.004253	7.20
Total	71	0.059065	100.00

**Table 6:** ANOVA for regression analysis.

Source	Abbreviation	DF (Degree of Freedom)	Seq SS	Contribution [%]
Regression		10	0.051528	87.24
pressure	p	1	0.002477	4.19
temperature	t	1	0.014008	23.72
velocity	v	1	0.00258	4.37
humidity	h	1	0.021013	35.5
pressure × temperature	p × t	1	0.002554	4.32
pressure × velocity	p × v	1	0.000189	0.32
pressure × humidity	p × h	1	0.006004	10.16
temperature × velocity	t × v	1	0.000002	0.00
temperature × humidity	t × h	1	0.0027	4.57
velocity × humidity	v × h	1	0.000002	0.00
Error		61	0.007537	12.76
Total		71	0.059065	100.00

## Data Availability

The data of this study are available from the corresponding author upon reasonable request.
